# Opposing roles for ADAMTS2 and ADAMTS14 in myofibroblast differentiation and function

**DOI:** 10.1002/path.6214

**Published:** 2023-11-06

**Authors:** Edward P Carter, Kubra K Yoneten, Nuria Gavara, Eleanor J Tyler, Valentine Gauthier, Elizabeth R Murray, Peter ten Dijke, Angus J Cameron, Oliver Pearce, Richard P Grose

**Affiliations:** ^1^ Centre for Tumour Biology, Barts Cancer Institute Queen Mary University of London London UK; ^2^ Department of Life Sciences University of Bath Bath UK; ^3^ Centre for Therapeutic Innovation, Faculty of Science University of Bath Bath UK; ^4^ Unitat de Biofísica i Bioenginyeria, Facultat de Medicina i Ciències de la Salut Universitat de Barcelona Barcelona Spain; ^5^ Centre for Tumour Microenvironment, Barts Cancer Institute Queen Mary University of London London UK; ^6^ Oncode Institute and Department of Cell and Chemical Biology Leiden University Medical Center Leiden The Netherlands

**Keywords:** pancreatic cancer, 3D *in vitro* models, cancer invasion, myofibroblast, cancer‐associated fibroblasts, protease, TGFβ, extracellular matrix, cellular cross talk

## Abstract

Crosstalk between cancer and stellate cells is pivotal in pancreatic cancer, resulting in differentiation of stellate cells into myofibroblasts that drives tumour progression. To assess cooperative mechanisms in a 3D context, we generated chimeric spheroids using human and mouse cancer and stellate cells. Species‐specific deconvolution of bulk‐RNA sequencing data revealed cell type‐specific transcriptomes underpinning invasion. This dataset highlighted stellate‐specific expression of transcripts encoding the collagen‐processing enzymes ADAMTS2 and ADAMTS14. Strikingly, loss of ADAMTS2 reduced, while loss of ADAMTS14 promoted, myofibroblast differentiation and invasion independently of their primary role in collagen‐processing. Functional and proteomic analysis demonstrated that these two enzymes regulate myofibroblast differentiation through opposing roles in the regulation of transforming growth factor β availability, acting on the protease‐specific substrates, Serpin E2 and fibulin 2, for ADAMTS2 and ADAMTS14, respectively. Showcasing a broader complexity for these enzymes, we uncovered a novel regulatory axis governing malignant behaviour of the pancreatic cancer stroma. © 2023 The Authors. *The Journal of Pathology* published by John Wiley & Sons Ltd on behalf of The Pathological Society of Great Britain and Ireland.

## Introduction

Treatment options are limited in pancreatic ductal adenocarcinoma (PDAC), due to late diagnosis and substantial tumour desmoplasia. Central to the dense desmoplasia are pancreatic stellate cells―tissue resident stromal cells that activate to become cancer‐associated fibroblasts (CAF) when exposed to the tumour milieu [1]. These activated stellate cells secrete high levels of extracellular matrix (ECM) [2], promote therapy resistance [3], facilitate cancer cell invasion [4], and contribute to the pool of CAFs within the tumour [5]. Consequently, stellate cells have received considerable attention as a potential therapeutic target [1]. However, efforts to target stellate cells have been complicated by CAF heterogeneity and conflicting functional roles.

Paradoxically, depletion of activated stellate cells leads to more aggressive tumours, shining a light on their tumour‐restrictive functions [6, 7]. The remarkable plasticity of stellate cells enables them to adopt multiple phenotypes when presented with distinct tumour microenvironmental cues [8, 9, 10]. Thus, unlocking the therapeutic potential of these cells demands a detailed understanding of their biology, to determine critical functional nodes that can be targeted, rather than focusing solely on depleting stellate cell number.

Using a heterospecific approach to generate chimeric PDAC spheres, followed by *post hoc* bioinformatic deconvolution, we established high‐fidelity transcriptional signatures of cancer and stellate cells in a 3D model of stellate‐led invasion. Interrogating these data to gain insight into cellular crosstalk in an invasive context, we showed that stellate cells specifically express the collagen processing enzymes ADAMTS2 and ADAMTS14. Cellular and biochemical analyses revealed that, while acting similarly in relation to collagen processing, these enzymes have directly opposed roles in myofibroblast differentiation, with both playing a critical role in regulating the bioavailability of transforming growth factor beta (TGFβ).

## Materials and methods

### Cell culture

All pancreatic cells were maintained free of antibiotics in DMEM:F12 medium (Sigma, Gillingham, UK) supplemented with 10% foetal bovine serum (FBS; Gibco, Loughborough, UK) at 37 °C in 5% CO_2_. Human Miapaca2 and Panc‐1 pancreatic cancer cell lines were a kind gift from Professor Hemant Kocher (Barts Cancer Institute, London, UK). Mouse pancreatic cancer cell lines R254 and DT6066 were derived from KPF and KPC pancreatic tumours, respectively [8, 11], and were a kind gift from Professor Kairbaan Hodivala‐Dilke (Barts Cancer Institute). PS1 stellate cells were a kind gift from Professor Hemant Kocher (Barts Cancer Institute). Mouse PSCs were isolated from wildtype C57BL/6 mice as described [8]. The 1089 myoepithelial cell line, a kind gift from Professor Louise Jones (Barts Cancer Institute), was maintained free of antibiotics in Ham's F12 medium (Gibco), supplemented with 10% FBS, 0.5 μg/ml hydrocortisone (Sigma), 10 ng/ml Epidermal Growth Factor (Sigma), and 5 μg/ml Insulin (Sigma).

### Spheroid assay

To form spheres, 20‐μl droplets containing a total of 1,000 cancer cells and stellate cells in a 1:2 ratio were prepared in methylcellulose (0.24%; M0512, Sigma) and plated on the underside of a tissue culture plate lid. After 24 h, the spheroids were collected and pelleted at 100 × *g* for 3 min, then resuspended in a gel mix consisting of 2 mg/ml Collagen I (354,236, Corning, Amsterdam, NL) and 17.5% Matrigel (354,234, Corning), prepared in culture medium and buffered to physiological pH with 1 m NaOH. Approximately six spheroids suspended in gel mix were added to precoated wells of a low‐attachment 96‐well plate and left to solidify at 37 °C before culture medium was added on top.

Spheroids were imaged using an Axiovert 135 (Carl Zeiss, Cambridge, UK) microscope and percentage invasive area quantified using ImageJ (Version 2.9.0, National Institutes of Health, Bethesda, MD, USA), using the equation:

$$\text{Invasive area}\;\left(\%\right)=\left(\left(\text{total area}-\text{central area}\right)/\text{central area}\right)\;\times 100.$$

### Migration assay

Approximately 700 cancer cells and 1,400 stellate cells were seeded in culture medium containing 1% FBS into the apical compartment of an Incucyte Clearview 96‐well plate (4582, Sartorius, Epsom, UK). Basolateral compartments were filled with culture medium containing 10% FBS, and plates were placed in an Incucyte S3 Imaging System (Sartorius). Images of the apical and basal sides of the 8‐μm porous membrane were captured every 4 h over 3 days. At each timepoint, migration was calculated as the percentage of cells present in the basal compartment compared to the total number of cells in both apical and basal compartments.

### siRNA transfection

Cells were transfected with siRNA using Lipofectamine 3000 (Invitrogen, Loughborough, UK). The indicated SMART Pool siGENOME siRNAs containing four siRNA duplexes were purchased from Horizon Bioscience (Cambridge, UK).

### TGFβ luciferase assay

SMAD‐Firefly luciferase reporter assay was performed as described previously [12]. 1089 myoepithelial cells were transfected with Renilla luciferase control plasmid (pRL, Promega, Southampton, UK) and reporter plasmid encoding SMAD‐Firefly luciferase [8] at a 3:2 ratio with a total DNA input of 500 ng, using Lipofectamine 3000 reagent (Invitrogen). Cells were serum‐starved overnight and subsequently stimulated with conditioned medium for 24 h. The Dual‐Glo luciferase assay system (Promega) was used to detect Firefly‐ and Renilla‐luciferase activities.

### CAGA‐eGFP reporter assay

Stellate cells (8,000) stably expressing a CAGA‐eGFP reporter construct [13] were embedded in collagen I:Matrigel hydrogels (consisting of 2 mg/ml collagen I and 17.5% Matrigel) in a 96‐well plate. After 72 h, gels were fixed with 10% neutral‐buffered formalin and imaged using an LSM880 Zeiss confocal microscope. CAGA‐eGFP fluorescence was then calculated per cell from max projection z‐stacks using ImageJ.

### Western blotting

Cells lysates were prepared in 50 mm Tris–HCl, 150 mm NaCl, 1% Nonidet P40 buffer supplemented with protease (Millipore, Livingston, UK), and phosphatase (Millipore) inhibitor cocktails. Proteins were separated on a 10% SDS‐PAGE gel, transferred onto a nitrocellulose membrane and blocked using 5% milk diluted in Tris‐buffered saline with 0.1% tween (TBST) before overnight incubation at 4 °C with a primary antibody (diluted 1:1,000 in 5% bovine serum albumin [BSA]/TBST). Membranes were then washed with TBST, then incubated for 1 h with a species‐appropriate HRP‐conjugated secondary antibody (diluted 1:5,000 in TBST). The horseradish peroxidase (HRP)‐linked secondary antibodies used were goat anti‐mouse (P0447, Dako, Ely, UK) and goat anti‐rabbit (P0448, Dako). Bands were visualised using Immobilon Forte Western HRP Substrate (Millipore) using an Amersham Imager 600 (GE Healthcare, Amersham, UK). Primary antibodies used were mouse anti‐αSMA (1:5000, M0851, Dako), rabbit anti‐fibulin 2 (1:1000, PA521640, Invitrogen), rabbit anti‐ADAMTS14 (1:1000, PA5103578, Invitrogen), and mouse anti‐HSC70 (1:5000, sc7298, Insight Biotechnology, Wembley, UK). Secondary antibodies used were HRP‐conjugated goat anti‐mouse (P0447, Dako) and HRP‐conjugated goat anti‐rabbit (P0448, Dako).

### Immunofluorescence and imaging

Cells were fixed with 4% formaldehyde, permeabilised with 0.1% Triton X‐100 for 15 min, and then blocked in 5% BSA/phosphate‐buffered saline (PBS) for 1 h. Cells were then incubated for 1 h with anti‐αSMA (M0851, Dako) primary antibody diluted 1:200 in 5% BSA/PBS. Subsequently, cells were incubated for 1 h with Alexa‐546 donkey anti‐mouse (A11003, Invitrogen) secondary antibody diluted 1:200 in 5% BSA/PBS before being mounted using MOWIOL solution. Where indicated, filamentous actin was labelled through incubation with Alexa‐647 phalloidin (Cell Signaling Technology, Danvers, MA, USA) prior to mounting.

Spheroids containing fluorescently labelled cells were fixed in 10% formalin before mounting in MOWIOL solution on slides with a raised border for coverslip spacing.

Collagen gels were fixed in 10% neutral‐buffered formalin and embedded in paraffin wax. Sections cut at 3‐μm were then stained for collagen fibres using PicroSirius Red by the BCI Pathology Core Facility, and imaged on a Pannoramic scanner (3DHISTECH, Budapest, Hungary).

2D immunofluorescence images were acquired using either a Zeiss LSM710 confocal microscope or INCA2200 high‐content microscope (GE Healthcare). 3D spheroid z‐stack images were acquired using a Zeiss LSM880 confocal microscope. Second harmonic images of collagen gels were acquired using a Leica SP8 DIVE multiphoton microscope (Leica, Milton Keynes, UK).

αSMA fibre intensity per cell, and collagen fibre brightness within collagen gels, were calculated as described previously (*n* ≥ 5 random fields of view per biological repeat) [8, 14].

### Plasmin activity assay

Cells were cultured in phenol red free culture medium for 48 h before supernatant was collected and incubated with 0.2 mm chromogenic plasmin substrate D‐Val‐Leu‐Lys 4‐nitroanilide dihydrochloride (Sigma). After 30 min, absorbance was recorded at 405 nm on a 96‐well microplate reader (Infinite F50, Tecan, Reading, UK).

### RNA sequencing

Spheroid‐containing gels were lysed in TRIzol solution (Sigma), and RNA was isolated by isopropanol precipitation. RNA was sequenced at the genomics core facility at Queen Mary University of London (QMUL). Library preparation was performed using an NEB Next Ultra II (E7645S, NEB, Hitchin, UK) kit, and run on an Illumina NextSeq 500 (150 cycles, Illumina, Cambridge, UK). Reads were aligned to a combined human and mouse genome (using Human Hg38 and mouse mm10) using the STAR aligner, then separated based on species. Ambiguous reads were discarded from further analysis. Differential analysis between cell types from the same species was performed using Partek Flow software (Version 10, Partek, Chesterfield, MO, USA). Genes with greater than 2‐fold difference, with a false discovery rate (FDR) <0.01, were considered significantly different between groups. Gene overrepresentation analysis was performed using WEB‐based gene set analysis toolkit platform (http://www.webgestalt.org/, accessed March 2020).

### Proteomics

Matrisomics was performed as described previously [15, 16], and in Supplementary materials and methods.

### Statistical analysis

Apart from analysis of RNA Seq and Matrisome data, all analysis was performed using GraphPad Prism (v. 9.0, Boston, MA, USA) with statistical tests indicated in the figure legends. For individual cell datasets, statistical analysis was performed on the averages of each biological repeat.

## Results

### Chimeric spheroids reveal invasive cancer and stellate cell transcriptomes

**Figure 1:**
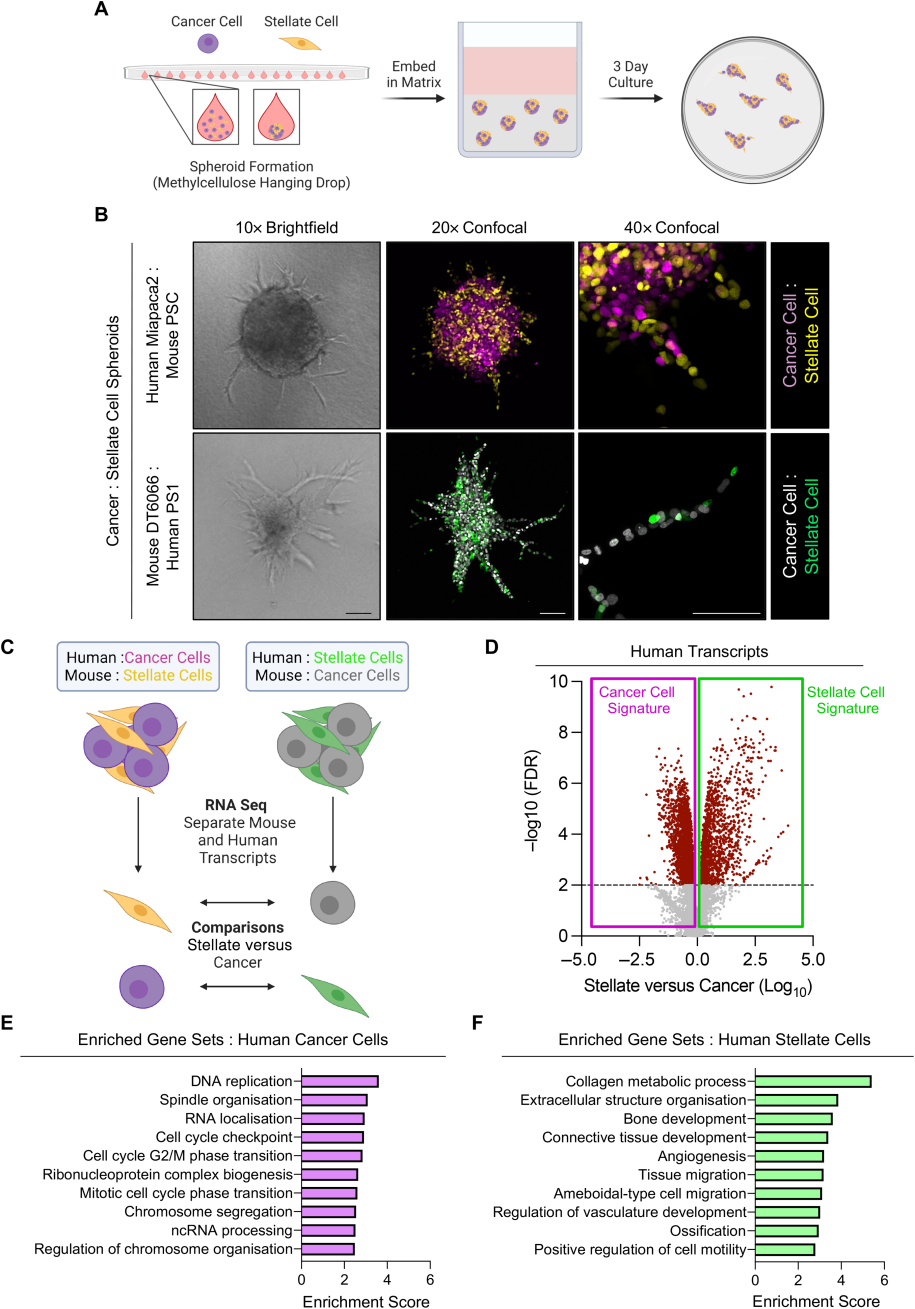
Chimeric spheres reveal cancer and stellate cell transcriptomes that underpin 3D invasion. (A) Schematic of spheroid invasion model. Stellate and cancer cells are formed into spheres using methylcellulose hanging drops, which are then placed in a 3D matrix and cultured for 3 days. (B) Brightfield and confocal images of chimeric spheres. Top panels, human cancer cells (Miapaca2; H2B‐RFP, purple) cocultured with mouse stellate cells (PSC; H2B‐GFP, yellow). Lower panels, mouse cancer cells (DT6066; H2B‐RFP, grey) mixed with human stellate cells (PS1; H2B‐GFP, green). Images representative of at least three biological replicates. Confocal images are representative collapsed z‐projections. Scale bar, 100 μm. (C) Schematic of transcriptomic approach. Spheroids are processed for bulk RNA sequencing and reads mapped to either human or mouse genome, providing cancer and stellate cell information. Cell information is then compared with the opposing cell type of the same species from corresponding spheroids. (D) Volcano plot of genes differentially regulated between stellate and cancer cells from human dataset. (E and F) Enriched gene sets in (E) human cancer cell and (F) human stellate cell datasets. Schematics created with BioRender.com.

We have previously described a 3D spheroid model of stellate‐led invasion using stellate and cancer cells combined into heterocellular spheres and embedded in a physiological matrix (Figure 1A) [8, 17, 18]. This model is highly tractable, allowing modulation of either cellular compartment, and is amenable to pharmacological interventions, with invasion and proliferation acting as objective, quantifiable readouts.

A challenge with heterocellular 3D models is identifying the cell type‐specific biology that underpins overall behaviour. Methods to isolate individual cell types do not necessarily maintain transcriptomic fidelity, particularly if lengthy digestion and separation steps are involved [19]. This can be overcome by using cell types from different species and deconvoluting bulk RNA sequencing data based on species, and thus cell type [20, 21].

We adopted this approach to explore cancer and stellate cell interactions in invasion, assembling chimeric spheroids from murine and human cancer and stellate cells. Intriguingly, while spheroids composed of human PDAC cells alone showed minimal invasion, spheres of murine PDAC cells could invade in the absence of stellate cells (supplementary material, Figure S1A). Nevertheless, both combinations generated spheres with invasive projections led by stellate cells (Figure 1B and supplementary material, Figure S1B), indicating that while murine PDAC cells could invade alone, stellate‐led invasion was the norm.

After 3 days culture, when invasive projections had formed, chimeric spheroids were harvested directly into RNA lysis buffer. Cell type‐specific information was then obtained from bulk RNA sequencing by matching sequencing reads to parent species, and thus cell type (Figure 1C). Comparing cancer cells with stellate cell signatures thus yielded cell type‐specific transcriptomes in an invasive context (Figure 1D and supplementary material, Figure S1C and Table S1). Gene overrepresentation analysis confirmed cell type‐specific information; cancer cell signatures were prominently enriched for genes involved in proliferation, while stellate cell signatures were enriched for genes related to invasion and matrix remodelling (Figure 1E,F and supplementary material, Figure S1D,E).

**Figure 2:**
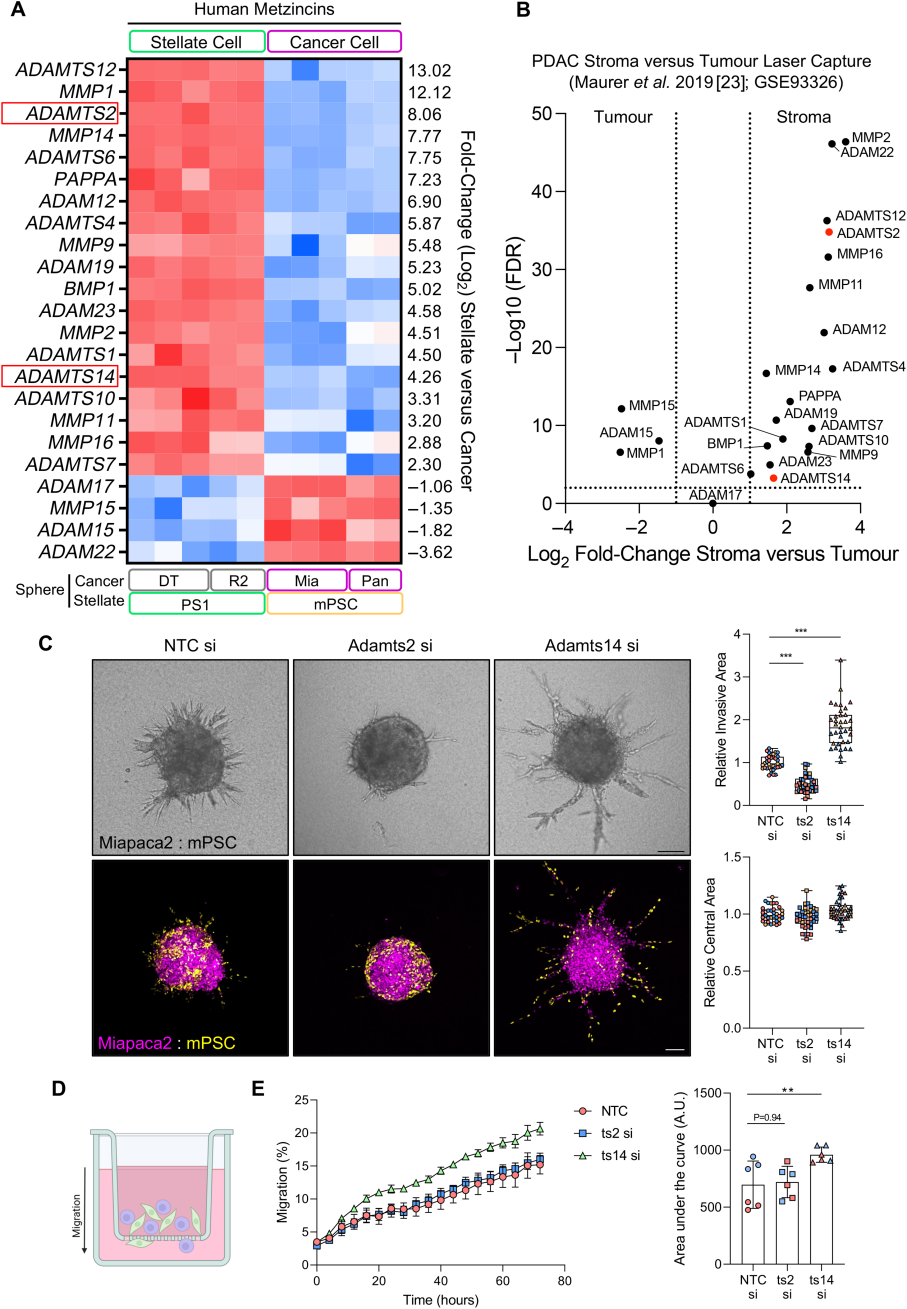
ADAMTS2 and ADAMTS14 have opposing roles in invasion. (A) Heatmap of metzincin expression in human dataset from chimeric spheroids. (B) Volcano plot of metzincins identified in A using RNA sequencing information from laser dissected PDAC tumour and stromal compartments. Data obtained from [23]. (C) Brightfield and confocal images and quantification of invasion and central area from Miapaca2 (H2B‐RFP, purple): mouse stellate cell (mPSC; H2B‐GFP, yellow) spheroids with siRNA knockdown of either *Adamts2* (ts2) or *Adamts14* (ts14) specifically in stellate cells. Scale bar, 100 μm. (D) Schematic of Boyden chamber migration assay. Fluorescently labelled Miapaca2 cancer and mouse stellate cells were added to the apical chamber and migration to the basal surface monitored. (E) Kinetics and area under the curve measurements of total cell migration (Miapaca2 + stellate cell) with stellate cell‐specific knockdown of either *Adamts2* or *Adamts14*. Images representative of three biological repeats. Confocal images are representative collapsed z‐projections. Individual colours represent distinct biological repeats. ****p < 0.0001, **p < 0.01, *p < 0.05. One‐way analysis of variance (ANOVA) with Dunnett's *post hoc* test. Schematic created with BioRender.com.

Of particular interest in the context of remodelling and invasion are the metzincin family of proteases, comprising both matrix metalloproteinases (MMPs) and a disintegrin and metalloproteinases (ADAMs). Members of this family are major invasion‐promoting proteases in PDAC [22] and our dataset highlights that the majority of these enzymes are produced by stellate cells (Figure 2A and supplementary material, Figure S2A). To establish the concordance of our dataset with human PDAC, we compared metzincin expression from our chimeric dataset with a published PDAC dataset comparing gene expression between laser‐dissected tumour and stromal compartments [23]. This confirmed that the majority of metzincin family members we identified are expressed in the PDAC stroma (Figure 2B), prompting us to interrogate the role of key family members in stellate‐cancer cell interactions.

### ADAMTS2 and ADAMTS14 have opposing roles in invasion

Compared to MMPs, the importance of ADAMs in regulating cancer invasion has received less attention. Interestingly, while ADAMTS12 was the highest expressed metzincin in the stellate compartment, silencing expression had no impact on spheroid invasion (data not shown). The role of collagen in PDAC has received considerable attention, with mature collagen fibres being shown to restrain PDAC progression [7, 24]. Collagen I is secreted as a procollagen trimer with subsequent cleavage of the N‐ and C‐termini allowing formation of mature collagen fibres. Downregulation of the C‐terminal collagen protease bone morphogenic protein 1 (BMP1) has been identified in PDAC, resulting in the formation of disorganised collagen fibres that facilitate progression [24]. We therefore focused on the two N‐terminal collagen processing enzymes identified from our data, ADAMTS2 and ADAMTS14, both of which are highly expressed in PDAC compared to normal tissue (supplementary material, Figure S2B).

*Adamts2*/*ADAMTS2* silencing in either mouse or human stellate cells significantly reduced stellate‐led invasion of cancer cells (Figure 2C and supplementary material, Figure S2C–E). Strikingly, however, knockdown of *Adamts14*/*ADAMTS14* in either mouse or human stellate cells enhanced invasion (Figure 2C and supplementary material, Figure S2C,F,G). No changes in sphere size were observed; indicating the result was not due to a change in overall cell number. An increase in cell migration following loss of *Adamts14* was confirmed by Boyden chamber migration assay (Figure 2D,E).

### ADAMTS2 and ADAMTS14 have opposing effects on myofibroblast differentiation

**Figure 3:**
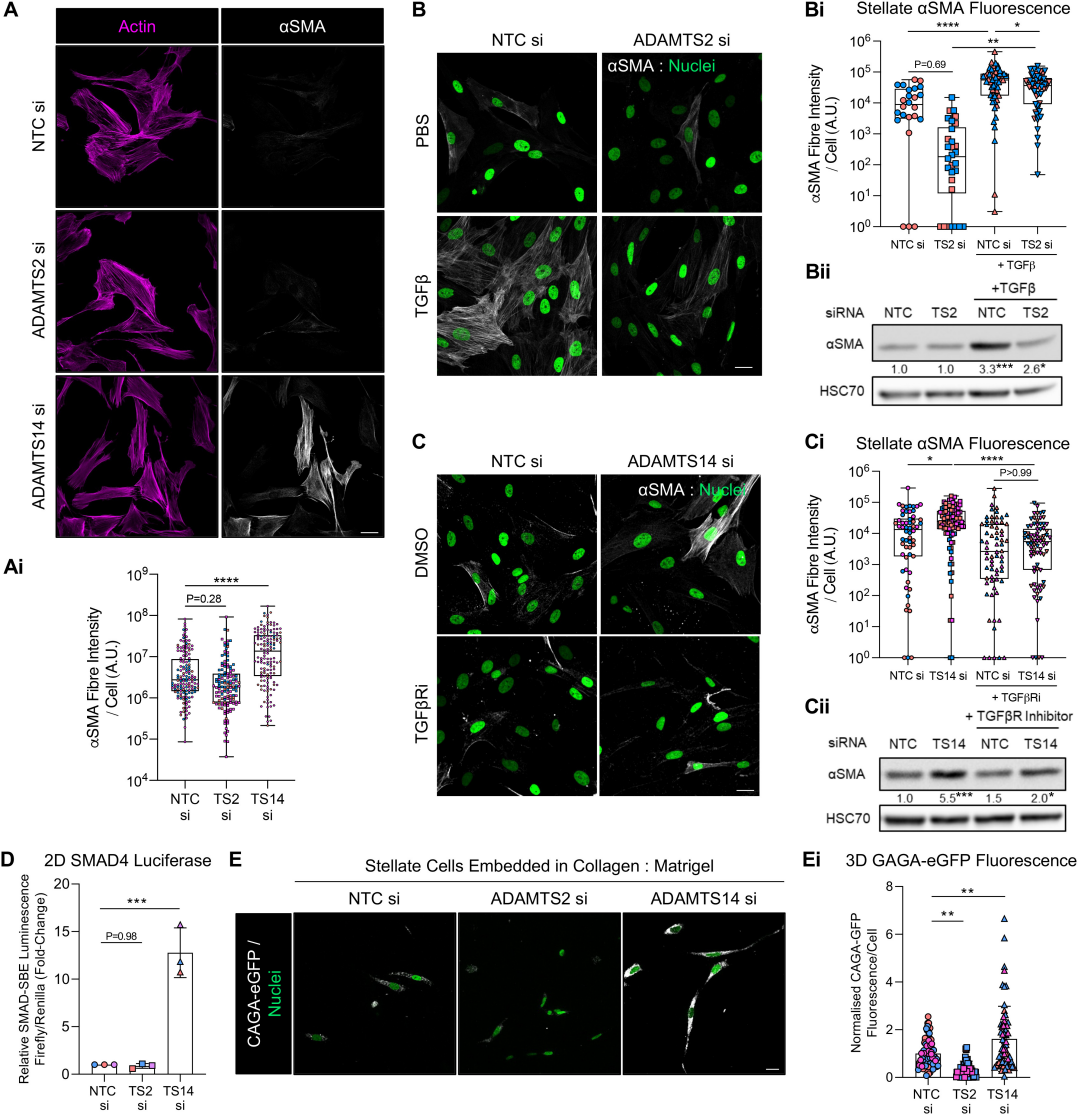
ADAMTS2 and ADAMTS14 have opposing roles in myofibroblast differentiation. (A) Confocal images of actin (purple) and αSMA (white) expression in stellate cells with knockdown of either *ADAMTS2* or *ADAMTS14*. Scale bar, 20 μm. (Ai) Quantification of αSMA fibre intensity per cell from A. (B) Confocal images of αSMA expression in human PS1 stellate cells following knockdown of *ADAMTS2* and stimulation with 5 ng/ml TGFβ for 48 h. Nuclei presented in green (H2B‐GFP). (Bi) Quantification of αSMA fibre intensity per cell from B. (Bii) Western blot for αSMA expression in human PS1 stellate cells following knockdown of ADAMTS2 and stimulation with 5 ng/ml TGFβ for 48 h. (C) Confocal images of αSMA in human PS1 stellate cells following knockdown of *ADAMTS14* and treatment with 10 μm SB431542 (TGFβR inhibitor) for 48 h. Nuclei presented in green (H2B‐GFP). (Ci) Quantification of αSMA fibre intensity per cell from C. (Cii) Western blot for αSMA expression in human PS1 stellate cells following knockdown of *ADAMTS14* and treatment with 10 μm SB431542 (TGFβR inhibitor) for 48 h. (D) SMAD reporter luminescence in 1,089 myoepithelial cells exposed for 24 h with conditioned medium from human PS1 stellate cells with indicated ADAMTS knockdown. Data presented as SMAD Firefly luminescence relative to control Renilla luminescence and normalised to conditioned medium from control stellate cells. (E) Representative z‐stack images of stellate cells with indicated knockdown and expressing a CAGA‐eGFP reporter construct embedded in collagen I: Matrigel hydrogels and cultured for 72 h. eGFP fluorescence presented in grey and cell nuclei in green. (Ei) Quantification of eGFP fluorescence from E. Data are presented as mean fluorescence intensity per cell normalised to respective background and control cells. Images representative of at least two biological repeats. Confocal image quantification preformed on at least five fields of view per sample. Individual colours represent distinct biological repeats. Densitometry of αSMA expression relative to HSC70 and normalised to respective control presented beneath blot. ****p < 0.0001, **p < 0.01, *p < 0.05. One‐way ANOVA with Dunnett's *post hoc* test. Scale bar, 20 μm.

Stellate‐led invasion is enhanced when stellate cells adopt a myofibroblastic phenotype, a key characteristic of which is the presence of α‐smooth muscle actin (αSMA) fibres [8]. Compared to control stellate cells, loss of *ADAMTS14* greatly enhanced αSMA fibre content, suggesting that lack of *ADAMTS14* drove stellate cells towards a myofibroblastic state (Figure 3A).

The principal growth factor regulating myofibroblast differentiation is TGFβ [25]. Exogenous TGFβ promotes αSMA expression and fibre formation, both of which were diminished significantly in stellate cells lacking *ADAMTS2* (Figure 3B). While loss of *ADAMTS14* promoted αSMA expression and fibre formation, co‐treatment with a TGFβ receptor (TGFβR) inhibitor significantly reduced this effect (Figure 3C).

In accord with an increased myofibroblastic state and increased TGFβ bioavailability, conditioned medium from stellate cells lacking *ADAMTS14* strongly drove expression of a SMAD4‐dependent luciferase reporter in recipient cells (Figure 3D). To explore the effect of *ADAMTS2/14* loss on TGFβ activity in 3D, we expressed a CAGA‐dependent fluorescent reporter, which drives expression of GFP in response to TGFβ [13], in stellate cells and embedded them in collagen I:Matrigel hydrogels. Consistent with 2D cell data, loss of *ADAMTS14* increased CAGA‐dependent fluorescence. Intriguingly, loss of *ADAMTS2* reduced CAGA‐dependent fluorescence (Figure 3E). Together, these data imply that ADAMTS2 and ADAMTS14 have opposing roles on myofibroblast differentiation, in a TGFβ‐dependent manner.

Somewhat surprisingly, when embedded in floating collagen gels, loss of either *ADAMTS2* or *ADAMTS14* in stellate cells reduced gel contraction (supplementary material, Figure S3A,B). Second harmonic generation (SHG) microscopy of collagen gels revealed a reduction in collagen remodelling following knockdown of either *ADAMTS2* or *ADAMTS14* (supplementary material, Figure S3C,D). The cell number within gels was consistent between conditions, suggesting that the effect was not a result of changes to proliferation (supplementary material, Figure S3E). These effects on collagen remodelling were confirmed through Picro‐Sirius Red imaging of sectioned collagen gels (supplementary material, Figure S3F).

### ADAMTS2 and ADAMTS14 regulate distinct matrisomal phenotypes

**Figure 4:**
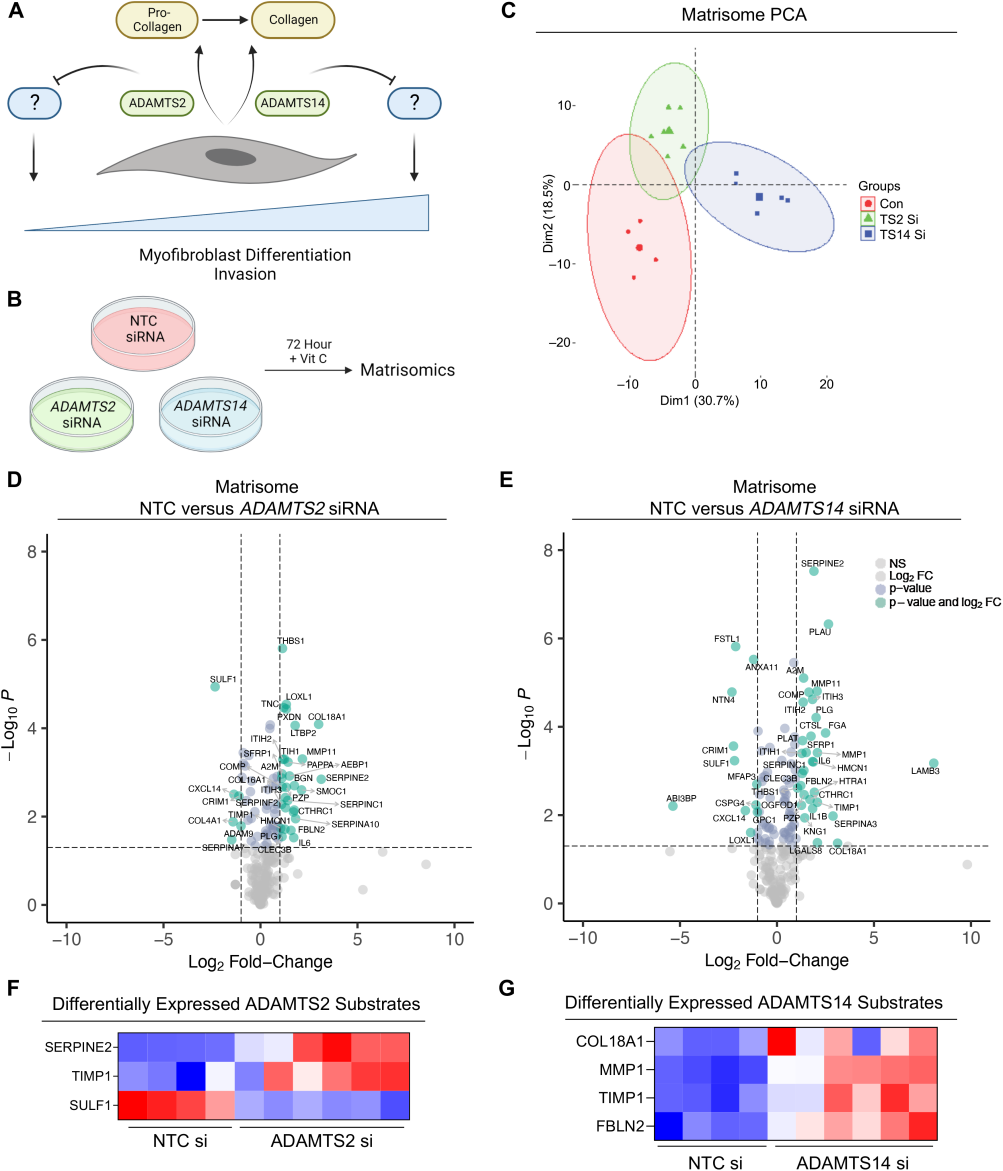
Loss ADAMTS2 and ADAMTS14 produce distinct matrisomes with enrichment of known substrates. (A) Schematic of proposed role of ADAMTS2 and ADAMTS14 in regulation of myofibroblast differentiation. (B) Schematic of matrisomic approach. (C) PCA plot of matrisome expression following knockdown of either *ADAMTS2* or *ADAMTS14* in human PS1 stellate cells. (D and E) Volcano plot of differentially expressed matrisome proteins following knockdown of either (D) *ADAMTS2* or (E) *ADAMTS14*. (F and G) Heatmaps of differentially expressed (F) ADAMTS2 and (G) ADAMTS14 substrates identified from matrisome data. Data obtained from at least two biological repeats analysed in technical duplicates. Schematic created with BioRender.com.

ADAMTS2 and ADAMTS14 play similar roles in collagen processing, suggesting that their roles in regulating myofibroblast differentiation are independent of their impact on collagen. We reasoned that their distinctive effects could be mediated through enzyme‐specific substrates (Figure 4A). Indeed, distinct repertoires of cleavage substrates have been reported for both enzymes [26], and therefore loss of either *ADAMTS2* or *ADAMTS14* could increase levels of the respective substrate(s) responsible for modulating myofibroblast differentiation.

As many of the identified substrates for these enzymes are ECM proteins [26], we analysed the matrisome of stellate cells following knockdown of either *ADAMTS2* or *ADAMTS14*. Loss of either protease generated distinct matrisome signatures (Figure 4B–E, supplementary material, Table S2), with greater changes observed following loss of ADAMTS14, likely reflective of differentiation to a myofibroblastic phenotype.

Comparison between our matrisome data and published ADAMTS2/14 substrates [26] identified significant enrichment of the ADAMTS2 substrates Serpin E2 and TIMP1 in stellate cells lacking *ADAMTS2* (Figure 4F). Furthermore, multiple ADAMTS14 substrates were significantly enriched in the matrisome of stellate cells following *ADAMTS14* knockdown, namely, COL18A1, MMP1, TIMP1, and fibulin 2 (*FBLN2*) (Figure 4G).

### The ADAMTS2 substrate SERPINE2 regulates myofibroblast differentiation

Having identified enrichment of the ADAMTS2 substrates TIMP1 and Serpin E2 in stellate cells with *ADAMTS2* knockdown (Figure 4F and supplementary material, Figure S4A), we next sought to identify if either was responsible for restraining myofibroblast differentiation. Knockdown of *Timp1* alongside *Adamts2* failed to rescue the reduced invasion caused by loss of *Adamts2* alone (supplementary material, Figure S4B,C). Loss of TIMP1 has previously been shown to promote a myofibroblastic phenotype [27], so its inability to affect invasion in this context was unexpected.

**Figure 5:**
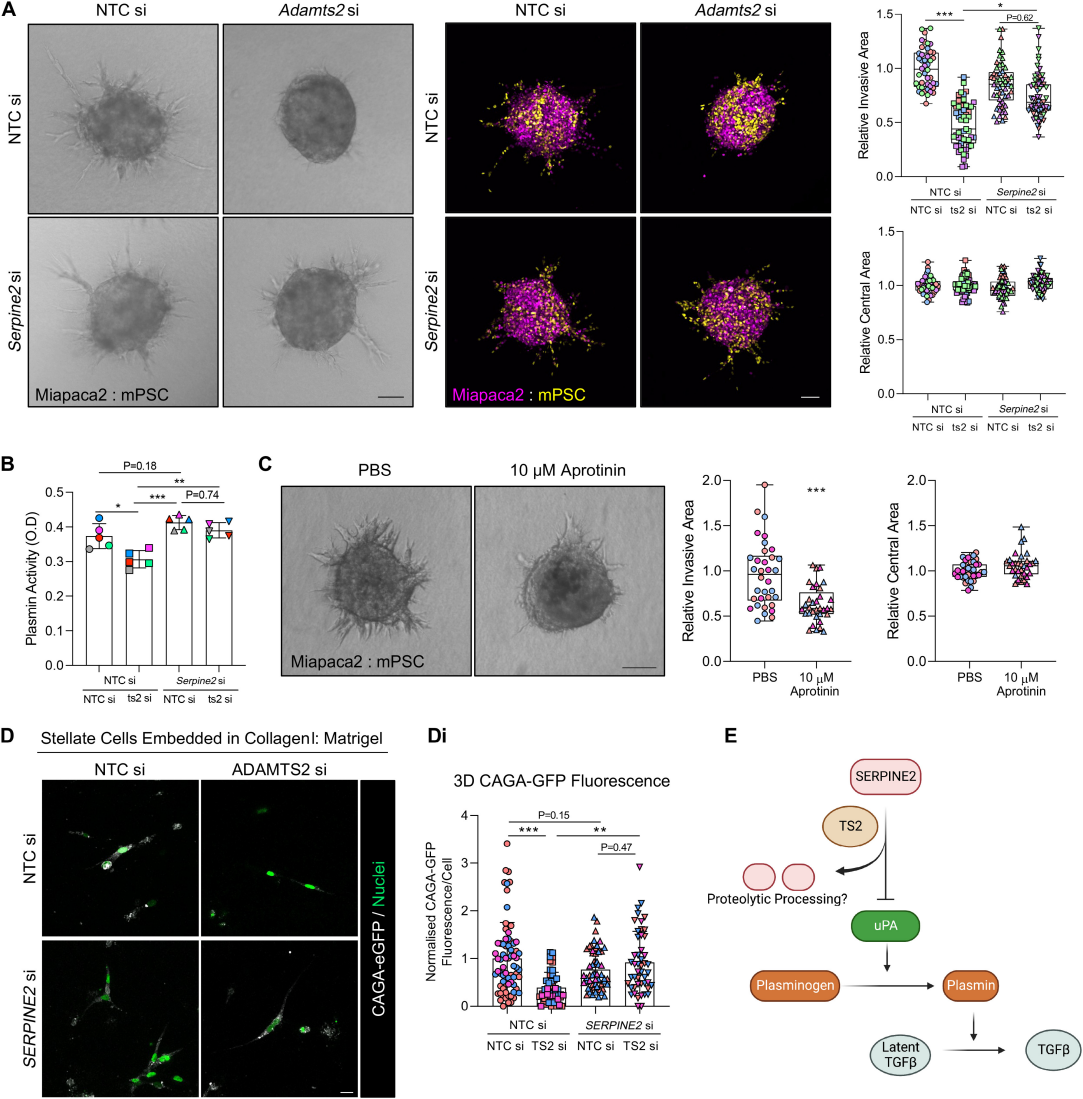
The ADAMTS2 substrate Serpin E2 regulates myofibroblast differentiation. (A) Brightfield and confocal images and quantification of invasion and central area from Miapaca2 (H2B‐RFP, purple): mouse stellate cell (mPSC; H2B‐GFP, yellow) spheroids with siRNA knockdown of *Adamts2* (ts2) with and without coknockdown of *Serpine2*. (B) Plasmin activity in mouse stellate cell supernatant 48 h following knockdown of *Adamts2* with and without coknockdown of *Serpine2*. (C) Brightfield images and quantification of invasion and central area of Miapaca2: mPSC spheroids treated with 10 μm aprotinin for 72 h. Scale bar, 100 μm. (D) Representative z‐stack images of stellate cells with indicated knockdown and expressing a CAGA‐eGFP reporter construct embedded in collagen I: Matrigel hydrogels and cultured for 72 h. eGFP fluorescence presented in grey and cell nuclei in green. Scale bar, 20 μm. (Di) Quantification of eGFP fluorescence from (D). Data are presented as mean fluorescence intensity per cell, normalised to respective background and control cells. Images representative of at least two biological repeats. Individual colours represent distinct biological repeats. ***p < 0.001, **p < 0.01, *p < 0.05. One‐way ANOVA with Dunnett's *post hoc* test. (E) Schematic proposing a role for ADAMTS2 and Serpin E2 in myofibroblast differentiation. ADAMTS2 degrades Serpin E2, which normally inhibits the action of Urokinase Plasminogen Activator (uPA). uPA catalyses the conversion of plasmin from plasminogen, which releases latent‐bound TGFβ. Loss of ADAMTS2 enhances Serpin E2 function, diminishing the release of active TGFβ. Schematic created with BioRender.com.

Concurrent knockdown of *SERPINE2/Serpine2* alongside *ADAMTS2/Adamts2* in either human or mouse stellate cells reversed the loss of invasion observed with *ADAMTS2/Adamts2* knockdown alone (Figure 5A and supplementary material, Figure S4D,E), suggesting Serpin E2 might be a key ADAMTS2 substrate in regulating invasion. Serpin E2 is a serine protease inhibitor that can modulate plasmin activity through inhibition of plasminogen activators [28]. In support of this, plasmin activity in stellate cell supernatant was reduced in cells lacking *Adamts2*, and activity was rescued upon concomitant knockdown of *Serpine2* (Figure 5B). Equally, treatment of spheres with the serine protease inhibitor aprotinin reduced invasion compared to control counterparts (Figure 5C).

A prominent role for plasmin in the tumour microenvironment is the proteolytic release of TGFβ from its latent form [29, 30, 31]. Accordingly, loss of *ADAMTS2* reduced CAGA‐dependent fluorescence, which was rescued with concomitant knockdown of *SERPINE2* in stellate cells embedded in collagen I: Matrigel hydrogels (Figure 5D). These data led to a proposed model where ADAMTS2 may facilitate TGFβ release through cleavage of the plasmin inhibitor, SERPINE2 (Figure 5E).

### ADAMTS14 regulates myofibroblast differentiation through fibulin 2

Loss of *ADAMTS14* produced a larger change in the stellate cell matrisome than *ADAMTS2* knockdown and increased the levels of the specific ADAMTS14 substrates COL18A1, MMP1, TIMP1, and fibulin 2 (Figure 4G and supplementary material, Figure S5A). To determine which might be responsible for the observed invasive phenotype, we performed an siRNA screen of all the upregulated matrisome proteins, in combination with *ADAMTS14* knockdown (supplementary material, Figure S5B,C). αSMA fibre intensity was assessed as a marker of myofibroblast differentiation (supplementary material, Figure S5C). Compared to *ADAMTS14* knockdown alone, combination with either *IL1B* or *KNG1* knockdown greatly increased αSMA staining. Of the known ADAMTS14 substrates, only concurrent knockdown of either *MMP1* or *FBLN2* was able to significantly reverse the effect of *ADAMTS14* knockdown alone (supplementary material, Figure S5C).

**Figure 6:**
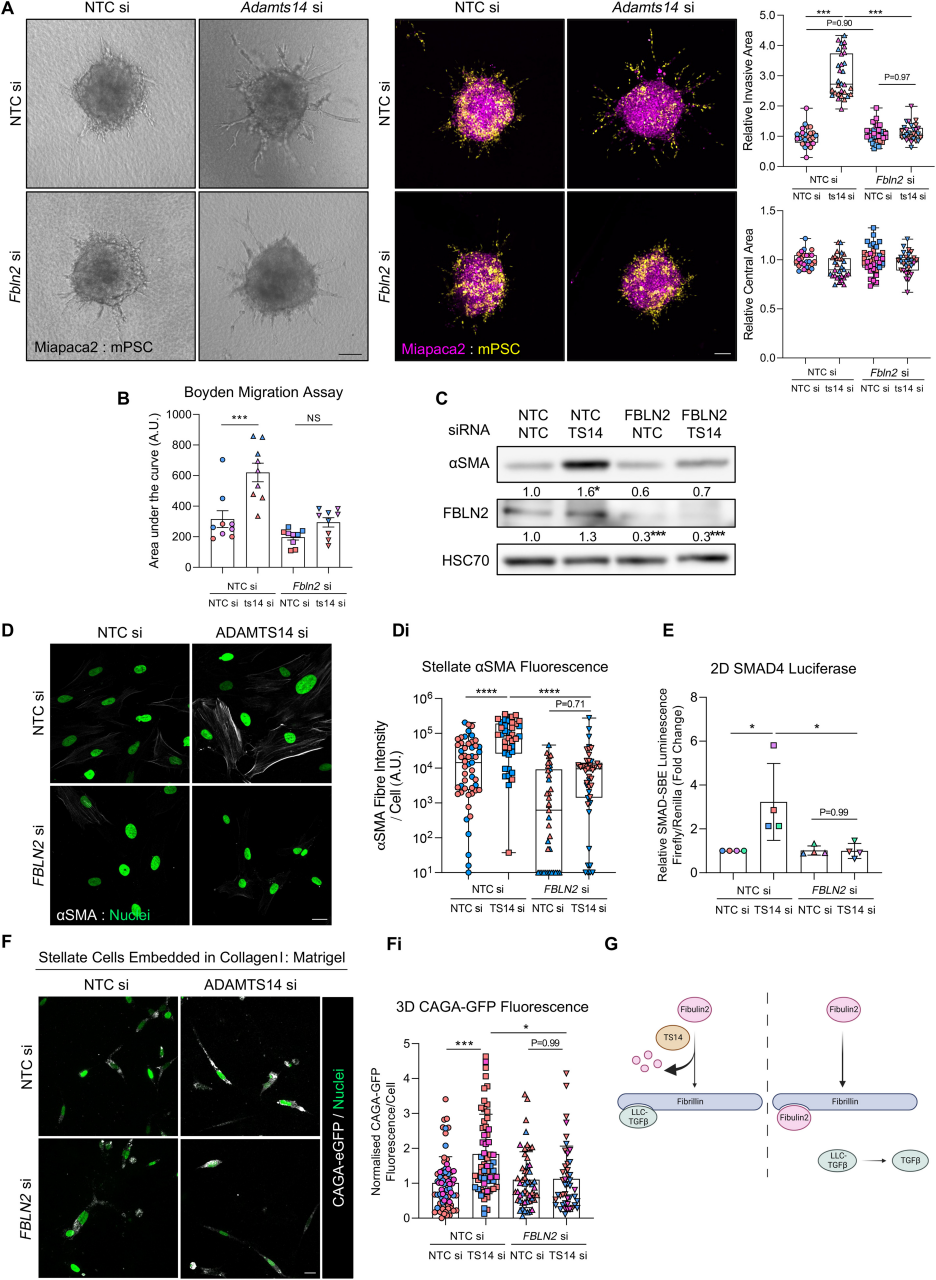
ADAMTS14 regulates myofibroblast differentiation through fibulin 2. (A) Brightfield and confocal images and quantification of invasion and central area from Miapaca2 (H2B‐RFP, purple): mouse stellate cell (mPSC; H2B‐GFP, yellow) spheroids with siRNA knockdown of *Adamts14* (ts14) with and without coknockdown of *Fbln2*. Scale bar, 100 μm. (B) Area under the curve analysis of cancer and stellate cell migration with mouse stellate cell knockdown of *Adamts14* alone or in combination with *Fbln2* knockdown. (C) Western blot for αSMA and fibulin 2 expression in human PS1 stellate cells with knockdown of *ADAMTS14* alone or in combination with *FBLN2* knockdown. Densitometry of αSMA and fibulin 2 expression relative to HSC70 and normalised to respective control is presented beneath the blot. (D) Confocal images of αSMA expression in stellate cells following knockdown of *ADAMTS14* alone or in combination with *FBLN2* knockdown. Scale bar, 20 μm. (Di) Quantification of αSMA fibre intensity per cell presented in (D). Confocal image quantification performed on at least five fields of view per sample. (E) SMAD reporter luminescence in 1,089 myoepithelial cells exposed for 24 h to conditioned medium from human PS1 stellate cells with *FBLN2* knockdown with and without coknockdown of *ADAMTS14*. Data presented as SMAD Firefly luminescence relative to control Renilla luminescence and normalised to conditioned medium from control stellate cells. (F) Representative z‐stack images of stellate cells with indicated knockdown and expressing a CAGA‐eGFP reporter construct embedded in collagen I: Matrigel hydrogels and cultured for 72 h. eGFP fluorescence presented in grey and cell nuclei in green. Scale bar, 20 μm. (Fi) Quantification of eGFP fluorescence from (F) Data are presented as mean fluorescence intensity per cell normalised to respective background and control cells. Images representative of at least two biological repeats. Individual colours represent distinct biological repeats. ****p < 0.0001, ***p < 0.001, *p < 0.05. One‐way ANOVA with Dunnett's *post hoc* test. (G) Schematic of possible role for ADAMTS14 and fibulin 2 in myofibroblast differentiation. Fibulin 2 and the TGFβ large latent complex compete for binding to fibrillin. In the absence of ADAMTS14, fibulin 2 outcompetes TGFβ large latent complex binding to fibrillin, releasing active TGFβ into the milieu. Schematic created with BioRender.com.

Despite perturbing αSMA fibre formation, dual knockdown of *MMP1* together with *ADAMTS14* in stellate cells was unable to prevent the enhanced invasive phenotype observed with loss of *ADAMTS14* alone (supplementary material, Figure S5D,E). Conversely, co‐knockdown of *FBLN2/Fbln2* in either human or mouse stellate cells blocked the invasive phenotype seen with *ADAMTS14*/*Adamts14* knockdown alone (Figure 6A and supplementary material, Figure S5F). Additionally, cell migration was also blocked by concomitant knockdown of both stellate cell *Adamts14* and *Fbln2* (Figure 6B).

In further support for a role for fibulin 2 in mediating the effects of ADAMTS14 on myofibroblast function, coknockdown abrogated the enhanced αSMA expression and fibre formation phenotype associated with *ADAMTS14* silencing (Figure 6C,D). Supporting a role for fibulin 2 in regulating the effects of ADAMTS14 on TGFβ activity, the enhanced TGFβ response to medium conditioned by cells lacking *ADAMTS14* was abrogated upon coknockdown of *FBLN2* (Figure 6E). Equally, enhanced CAGA‐dependent fluorescence observed from stellate cells lacking *ADAMTS14* and embedded in collagen I: Matrigel was reduced with concomitant knockdown of *FBLN2* (Figure 6F). Taken together, these data implicate ADAMTS14 as a regulator of TGFβ bioavailability, mediated through fibulin 2 (Figure 6G).

## Discussion

Stromal targeting in cancer requires knowledge regarding functions of the various stromal constituents, to ensure preferential targeting of protumoural axes over tumour‐restrictive ones. 3D model systems provide an excellent environment to dissect stromal biology, by faithfully recapitulating cell–cell and cell–matrix interactions in a simplified but highly tractable setting [19]. Our chimeric model of cancer/stellate interactions has captured the transcriptional profile of both cell types, providing cell type‐specific data from a 3D invasive context. This dataset serves as a useful resource to interrogate cell type‐specific functions and critical cell–cell signals required for invasion that can be exploited therapeutically.

We have focussed on protease expression in both cell types, due to their importance in invasion. Cell type‐specific expression of proteases in the tumour environment can be difficult to discern, due to their secreted nature. Our data demonstrate that stellate cells are the major source of these proteases.

The collagen‐processing enzymes ADAMTS2 and ADAMTS14 were highly enriched in our dataset, and loss of either perturbed collagen remodelling by stellate cells. Mutations in ADAMTS2 account for a dermatosparatic variant of Ehlers–Danlos syndrome, characterised by fragile skin as a consequence of impaired collagen processing [32]. *Adamts2* knockout mice display the same fragile skin condition but still exhibit some processed collagen in the dermis, owing to partial redundancy through Adamts14 [33]. Tissue‐specific expression and partial redundancy between the procollagen N‐endopeptidase family, which also includes ADAMTS3, explains why loss of ADAMTS2 does not have a more global effect on collagen‐rich tissues [33]. Fibroblasts derived from Ehlers–Danlos syndrome tissue lack the ability to contract collagen gels, due to downregulation of collagen‐interacting proteins such as integrin α2β1 [34, 35]. This may explain why loss of ADAMTS2 or ADAMTS14 failed to contract collagen gels in our context.

ADAMTS2 and ADAMTS14 exhibited remarkably divergent roles on myofibroblast differentiation, implicating crucial collagen‐independent roles for these enzymes. Indeed, alternative substrates for the ADAMTS family have been identified, demonstrating their much broader roles in matrix regulation [26]. For instance, ADAMTS3 is necessary for lymphangiogenesis through proteolytic processing of VEGF‐C [36], and suppresses early breast cancer invasion through degradation of fibronectin [37].

Our matrisomics approach revealed elevated levels of known ADAMTS2 and ADAMTS14 substrates, with further mechanistic studies implicating their influence on stellate‐led invasion. A significant increase in serpin E2, a serine protease inhibitor and known ADAMTS2 substrate [26], following *ADAMTS2* silencing, implicated this as a key mediator of the antiinvasive phenotype. Serpin E2 blocks the activity of Urokinase Plasminogen Activator (uPA) [28], which in turn cleaves Plasminogen to yield active Plasmin [38]. Plasmin plays an important role in the tumour microenvironment by proteolytically releasing active TGFβ from its latent complex [29, 30, 31]. Free TGFβ drives stellate cells towards a proinvasive myofibroblastic phenotype [25]. Given that loss of ADAMTS2 perturbs TGFβ activity, we propose that ADAMTS2 regulates myofibroblast differentiation though proteolytic processing of serpin E2. Although whether this is through degradation of serpin E2 or generation of a product with altered bioavailability remains to be determined. In the absence of ADAMTS2, serpin E2 levels increase, which in turn blocks the activity of uPA. This reduces the activation of Plasmin, potentially preventing the release of TGFβ (Figure 5E).

To interrogate the anti‐invasive effect of ADAMTS14, we focussed on determining key substrates that might mediate the promyofibroblastic phenotype seen upon *ADAMTS14* silencing. Although we focussed on targets that blocked αSMA fibre formation following *ADAMTS14* silencing in our combination siRNA screen, the validity of the screen was confirmed by enhanced αSMA fibre formation following concomitant loss of *IL1B*, which is known to antagonise the effects of TGFβ [25].

More importantly, the screen identified the ADAMTS14 substrate fibulin 2 as a potential mediator of the phenotype observed upon ADAMTS14 loss. Fibulin2 regulates the availability of matrix‐bound TGFβ by competing with the large latent complex of TGFβ for binding to the matrix component Fibrillin [39]. The importance of this matrix sink for TGFβ regulation is exemplified in Marfan syndrome, which is caused by mutations in the binding site for latent TGFβ on Fibrillin and is characterised by hyperactive TGFβ signalling [40]. Fibrillin is also a major component of the pancreatic tumour microenvironment [41]. This leads us to hypothesise that ADAMTS14 regulates myofibroblast differentiation by modulating fibulin 2 proteolysis. In the absence of ADAMTS14, fibulin 2 levels are increased, and fibulin 2 in turn outcompetes latent TGFβ for binding to Fibrillin, facilitating the release of active TGFβ into the tumour milieu (Figure 6G). An alternative hypothesis is that fibulin 2 acts as a cofactor for ADAMTS14, modulating its function, similar to what has been described for fibulin 1 and ADAMTS1 [42].

Stellate cells are remarkably plastic and will adopt different states, depending on extracellular cues [10], with discrete tumoural compartment signalling, further refining cellular phenotypes [25]. Together, our data reveal that ADAMTS2 and ADAMTS14 can differentially regulate the myofibroblastic differentiation of stellate cells, potentially through differential regulation of TGFβ bioavailability. Thus, these enzymes or their substrates may present novel opportunities for biomarker development and innovative angles for stromal targeting in PDAC. This work also demonstrates a broader role for these proteases in mediating cellular biology and highlights the complex regulation of the extracellular matrix.

## Author contributions statement

EPC and RPG conceived and designed the study. EPC, KKY, NG, EJT, VG and ERM acquired and analysed data. PD provided the CAGA‐eGFP construct. All authors contributed to interpretation of the data. EPC and RPG wrote the article with review and approval of all authors.

## Supporting information


Supplementary materials and methods
**Figure S1.** Chimeric spheres as a tool to uncover cancer and stellate cell transcriptomes in a 3D, invasive, environment
**Figure S2.** ADAMTS2 and ADAMTS14 are enriched in pancreatic cancer and have opposing roles in invasion
**Figure S3.** Stellate‐derived ADAMTS2 and ADAMTS14 both contribute to collagen remodelling
**Figure S4.** Serpin E2, and not TIMP1, contributes to observed phenotype following loss of ADAMTS2
**Figure S5.** siRNA screen reveals fibulin 2 as a mediator of ADAMTS14 function
**Table S1.** Differential expression between stellate and cancer cell transcriptomes from chimeric 3D invasive spheroids
**Table S2.** Stellate cell matrisome following loss of ADAMTS2 or ADAMTS14
**Table S3.** Primer sequences used

## Data Availability

The chimera RNA sequencing data are publicly available on the Gene Expression Omnibus archive (GSE225161; https://www.ncbi.nlm.nih.gov/geo/query/acc.cgi?acc=GSE225161). All further datasets generated and analysed are available upon request.
