# Supplementary material for: Opposing roles for ADAMTS2 and ADAMTS14 in myofibroblast differentiation and function
Source: J Pathol. 2023 Nov 6;262(1):90–104. doi: 10.1002/path.6214 (PMC10953099; doi:10.1002/path.6214)
Supplement: Supplementary file 1 — Supplementary materials and methods Figure S1. Chimeric spheres as a tool to uncover cancer and stellate cell transcriptomes in a 3D, invasive, environment Figure S2. ADAMTS2 and ADAMTS14 are enriched in pancreatic cancer and have opposing roles in invasion Figure S3. Stellate‐derived ADAMTS2 and ADAMTS14 both contribute to collagen remodelling Figure S4. Serpin E2, and not TIMP1, contributes to observed phenotype following loss of ADAMTS2 Figure S5. siRNA screen reveals fibulin 2 as a mediator of ADAMTS14 function Table S1. Differential expression between stellate and cancer cell transcriptomes from chimeric 3D invasive spheroids Table S2. Stellate cell matrisome following loss of ADAMTS2 or ADAMTS14 Table S3. Primer sequences used [file PATH-262-90-s001.zip › path6214-sup-0001-SuppMatMeth,FiguresS1-S5,TablesS1-S3/path6214-sup-0001-SuppMatMeth,FiguresS1-S5,TablesS1-S3.docx.docx]

**Opposing roles for ADAMTS2 and ADAMTS14 in myofibroblast differentiation and function**

EP Carter *et al. J Pathol* <https://doi.org/10.1002/path.6214>

**Supplementary materials and methods**

**Supplementary Figures S1–S5**

**Supplementary Tables S1,S2 are provided as separate Excel files**

**Supplementary Table S3**

Reference numbers refer to the main text list

**Supplementary materials and methods**

***Collagen gel contraction assay***

Stellate cells (50,000) were cast into 3 mg/ml Collagen I gels (Corning, Corning, NY, USA; 354236) prepared with 10x DMEM (D2429, Sigma, Gillingham, UK) and buffered to physiological pH with 1- NaOH. Gels were placed into wells of a 24-well tissue culture plate and solidified at 37°C for 1 h before culture medium was added on top. After 24 h, gels were released and allowed to float in culture medium. Gels were imaged after 72 h and the level of contraction calculated by comparing the area of the gel to the area of the well.

***RNA extraction and RT-qPCR***

RNA was extracted using the Monarch Total RNA Miniprep kit (T2010, New England Biolabs, NEB, Hitchin, UK), and reverse transcription was performed using LunaScript RT SuperMix (E3010, NEB). qPCR samples were prepared using Luna Universal qPCR Master Mix (M3003, NEB) and analysed using a Step One Plus Instrument (Applied Biosystems, Warrington, UK) with recommended cycle conditions. Primers used are listed in supplementary material, Table S3.

***Lentivirus production***

Lentiviral particles were generated by cotransfecting HEK293T cells with 3.25 μg pCMVR8.2 (Addgene, Watertown, MA, USA; #12263) and 1.7 μg pMD2.G (Addgene #12259) packaging plasmids, and 5 μg of either H2B-GFP (Addgene #11680), H2B-RFP (Addgene #26001), or CAGA-eGFP[13] plasmids using FuGENE transfection reagent (Promega, Southampton, UK). Virus-containing supernatant was harvested 48 h after transfection and stored at –80 °C.

For viral transduction, cells were cultured in viral supernatant for 24 h, after which the culture medium was replaced. Successfully transduced cells containing fluorescent reporters were isolated using a BD FACS Aria Fusion cell sorter (BD, Wokingham, UK).

**Proteomics**

Cells were cultured in phenol red free DMEM:F12 medium (Gibco, Loughborough, UK) supplemented with 10% FBS (Gibco, Gaithersburg, MD, USA) and medium supplemented with 50 µg/ml L-ascorbic acid (Sigma, St. Louis, MO, USA) for 48 h to stimulate ECM production. Cell lysates were prepared in 8 M urea supplemented with 100 mM Na_3_VO_4_, 500 mM NaF, 1 M β-Glycerol Phosphate, and 250 mM Na_2_H_2_P_2_O_7_.

Proteins were reduced with 25 mM DTT and alkylated with 40 mM iodoacetamide prior to the addition of 500 U/µl PNGase F (P0704, NEB) to remove N-glycosylations. Proteins were then digested with LysC (1.6 µg/sample) (90051, ThermoFisher Scientific, Loughborough, UK) for 2 h and further digested using immobilised-trypsin beads (40 µl beads/250 µg protein) (10066173, ThermoFisher Scientific) for 16 h.

Peptides were desalted using C-18 tip columns (Glygen, Ellicott City, MD, USA). Desalted samples were vacuum-dried and stored at –20 ºC. Dried peptide mixtures were dissolved in 0.1% trifluoroacetic acid and analysed using a nanoflow ultra-high pressure liquid chromatography system (NanoACQUITY UPLC System, Waters, Wilmslow, UK) coupled to an LTQ XL™ Linear Ion Trap mass spectrometer (ThermoFisher Scientific) at the Mass Spectrometry Core Facility, Barts Cancer Institute (UK).

Peptide identification was performed by screening the raw data against the SwissProt database (v. 2013-2014) restricted to human entries using the Mascot search engine (v. 2.5.0, Matrix Science, London, UK) with the following parameters: trypsin as digestion enzyme (with up to two missed cleavages), carbamidomethyl (C) as a fixed modification, and *N*-terminal pyroglutamate (pyroGlu), Oxidation (M), and Phospho (STY) as variable modifications; 5 ppm as peptide mass tolerance, ±0.8 Da as fragment mass tolerance. A MASCOT score cutoff of 50 was used to filter false-positive detection to an FDR <1%. Matrisome proteins were identified using the matrisome annotator tool (http://matrisomeproject.mit.edu/analytical-tools/matrisome-annotator/, accessed July 2021). *t*-tests were used to compare differences in protein abundance between samples with a *p*-value of <0.05 and a log_2_ fold-change >1 considered significant.

**Supplementary Figures S1–S5**

**
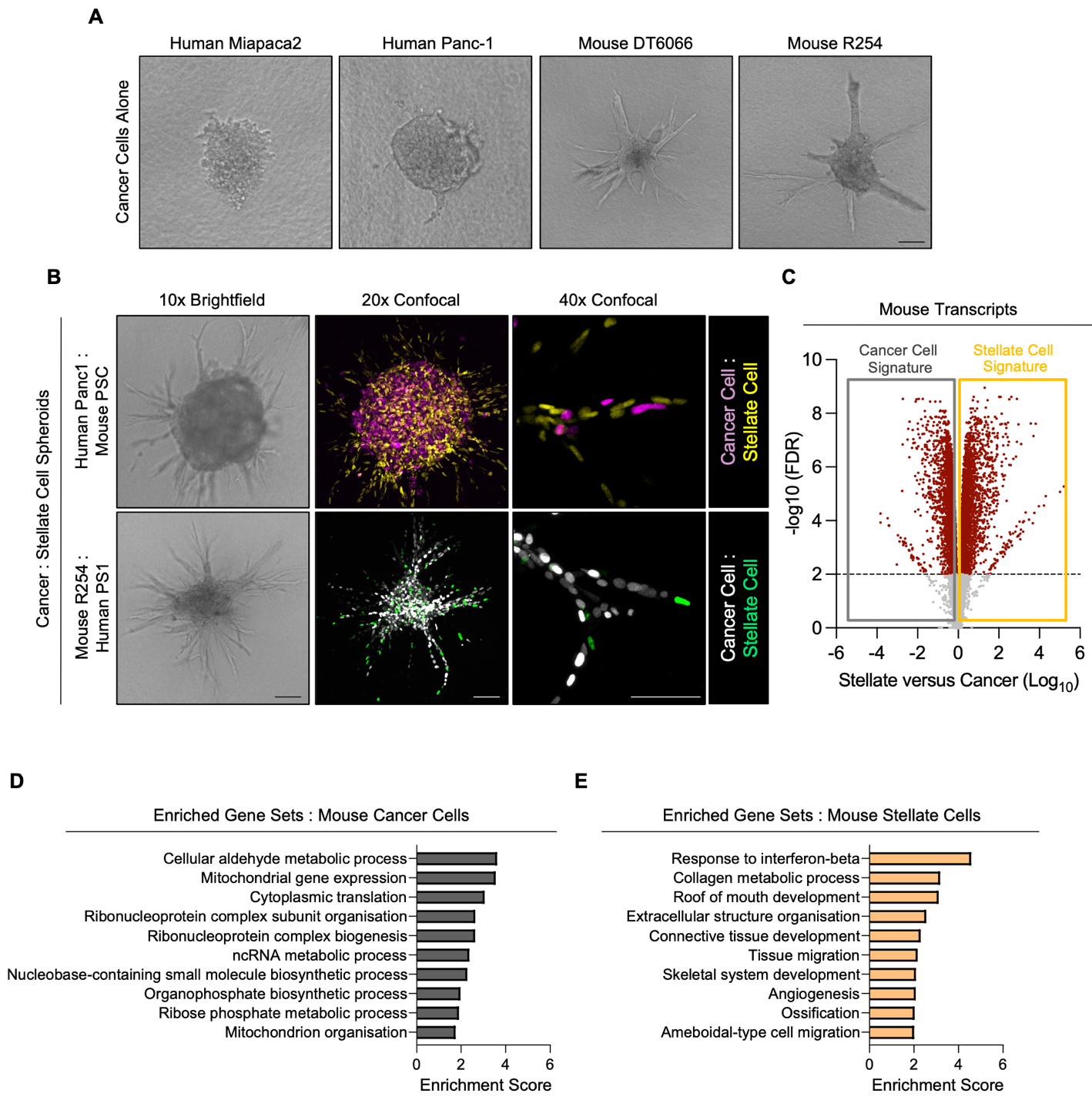
**

**Figure S1.** **Chimeric spheres as a tool to uncover cancer and stellate cell transcriptomes in a 3D, invasive, environment.** (A) Brightfield images of spheres composed solely of indicated cancer cells. (B) Brightfield and confocal images of chimeric spheres. Top panels, human cancer cells (Panc-1; H2B-RFP, purple) mixed with mouse stellate cells (PSC; H2B-GFP, yellow). Lower panels, mouse cancer cells (R254; H2B-RFP, grey) mixed with human stellate cells (PS1; H2B-GFP, green). Images representative of at least three biological replicates. Confocal images are representative collapsed z-projections. Scale bar, 100 μm. (C) Volcano plot of genes differentially regulated between stellate and cancer cells from murine dataset. (D,E) Enriched gene sets in (D) murine cancer cell and (E) murine stellate cell datasets.


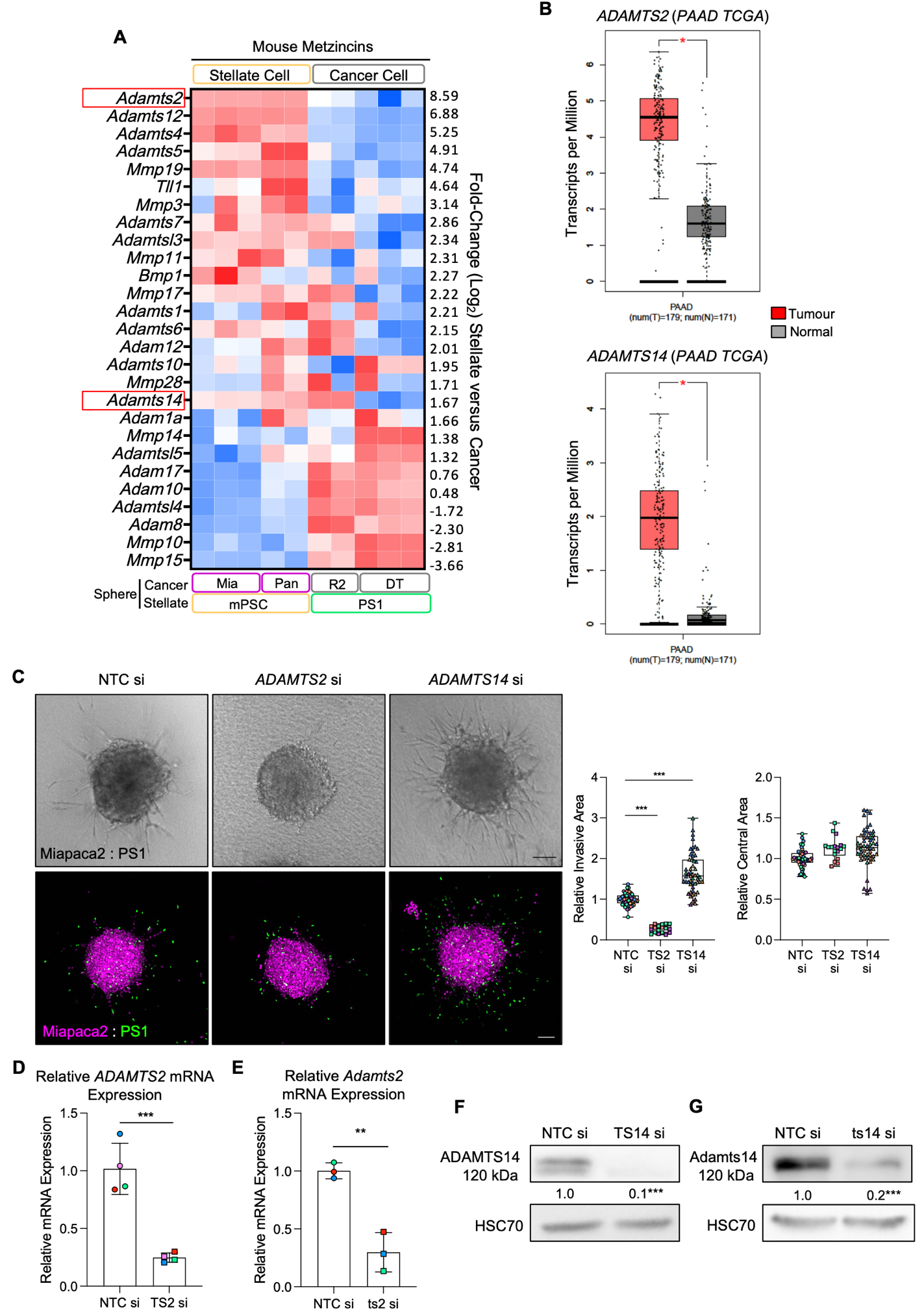


**Figure S2.** **ADAMTS2 and ADAMTS14 are enriched in pancreatic cancer and have opposing roles in invasion.** (A) Heat map of metzincin expression in murine dataset from chimeric spheroids. (B) *ADAMTS2* and *ADAMTS14* expression in PDAC and normal tissue, data obtained from TCGA. (C) Brightfield and confocal images and quantification of invasion and central area from Miapaca2 (H2B-RFP, purple): human PS1 stellate cell (H2B-GFP, green) spheroids with siRNA knockdown of either *ADAMTS2* (TS2) or *ADAMTS14* (TS14) specifically in stellate cells. Images representative of three biological repeats. Individual colours represent distinct biological repeats. Confocal images are representative collapsed z-projections. Scale bar, 100 μm. (D,E) Human *ADAMTS2* (D) and murine *adamts2* (E) expression in human PS1 and mouse stellate cells, respectively, following siRNA knockdown of *ADAMTS2* (TS2) or *Adamts2* (ts2). (F,G) Western blot for (F) human ADAMTS14 and (G) mouse Adamts14 expression in human PS1 and mouse stellate cells, respectively, following siRNA knockdown of *ADAMTS14* (TS14) or *Adamts14* (ts14). Densitometry of ADAMTS14 expression relative to HSC70 and normalised to respective control is presented beneath the blot. *** *p <* 0.01. One-way ANOVA with Dunnett’s *post hoc* test.


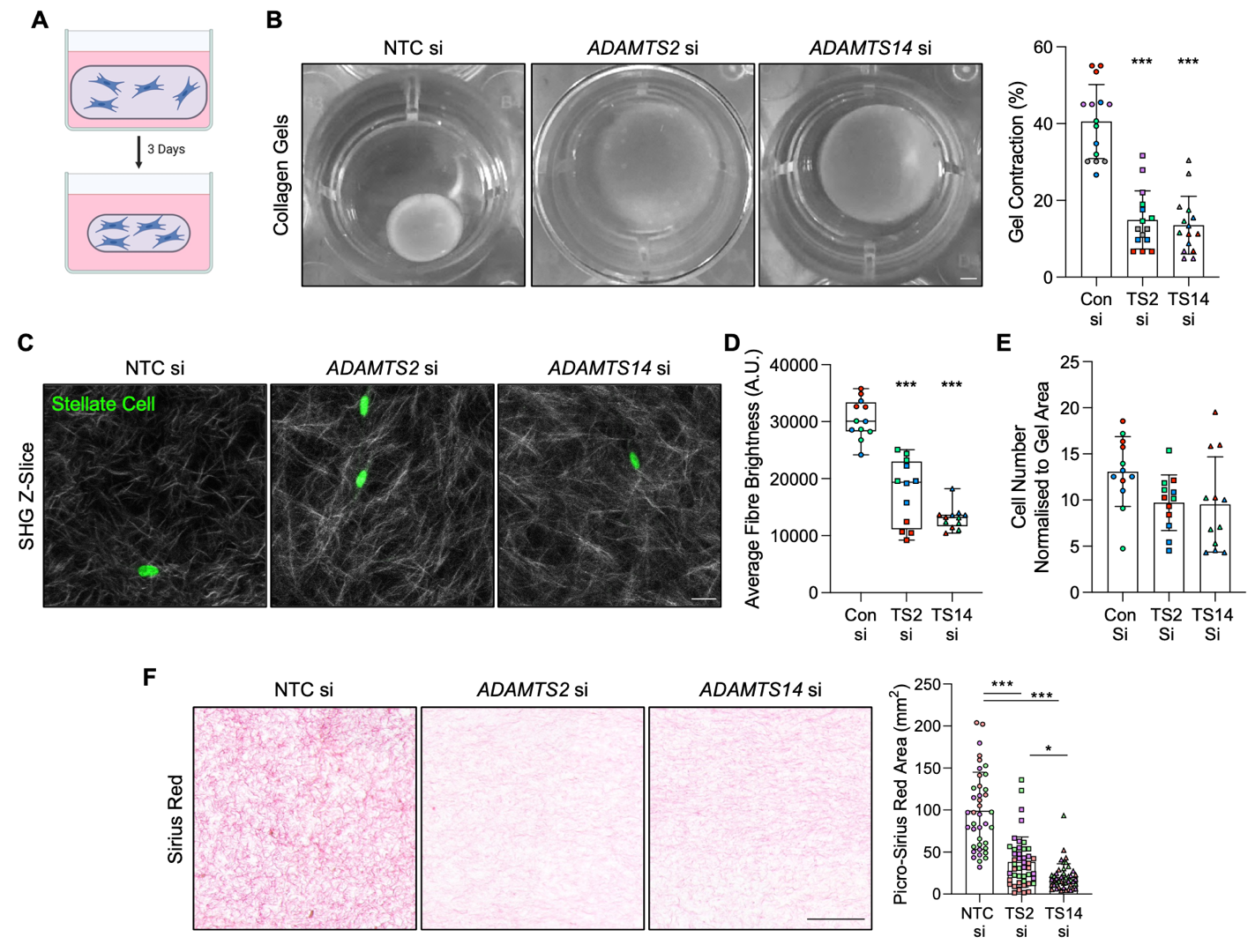


**Figure S3. Stellate-derived ADAMTS2 and ADAMTS14 both contribute to collagen remodelling.** (A) Schematic of collagen gel contraction assay. (B) Brightfield images and quantification of collagen gel contraction following siRNA knockdown of either *ADAMTS2* or *ADAMTS14* in embedded human PS1 stellate cells and cultured for 3 days. Images representative of at least three biological repeats performed in triplicate. Scale bar, 1 mm. (C) Representative single z-slices of second harmonic generation (SHG) microscopy of collagen gels presented in B. Stellate cell nuclei presented in green (H2B-GFP). Images captures from central portion of gel. Scale bar, 20 μm. (D) Quantification of relative cell number of stellate cells in collagen gels. Nuclei counted from four random collapsed z-projections per gel and adjusted for gel area at time of imaging. (E) Quantification of average fibre brightness from SHG images. (F) Picro-Sirius Red images and quantification of sections from collagen gels shown in B). Scale bar, 100 μm. **** *p <* 0.0001, *** *p <* 0.001, **p <* 0.05. One-way ANOVA with Dunnett’s *post hoc* test. Individual colours representative of distinct biological repeats.


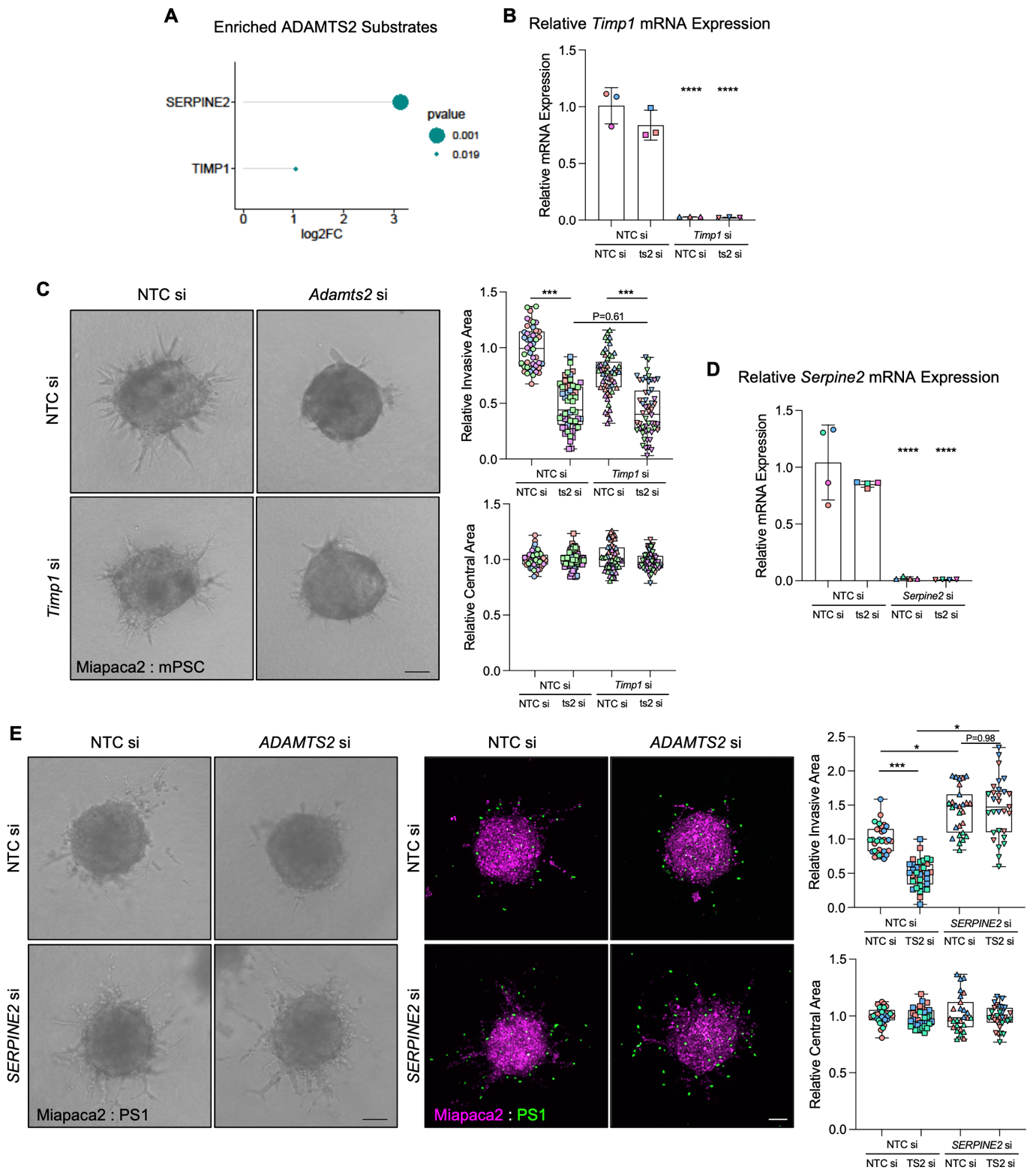


**Figure S4.** **Serpin E2, and not TIMP1, contributes to observed phenotype following loss of ADAMTS2.** (A) Lollipop plot of enriched ADAMTS2 substrates from matrisome data. (B) RT-qPCR for *Timp1* expression in murine stellate cells following siRNA knockdown. (C) Brightfield images and quantification of invasion and central area from miapaca2: mPSC spheroids with siRNA knockdown of *Adamts2* (ts2) with and without co-knockdown of *Timp1*. D) RT-qPCR for *Serpine2* expression in murine stellate cells following siRNA knockdown. (E) Brightfield and confocal images and quantification of invasion and central area from Miapaca2 (H2B-RFP, purple): human PS1 stellate cell (H2B-GFP, green) spheroids with siRNA knockdown of *ADAMTS2* with and without co-knockdown of *SERPINE2*. Images represent at least three biological repeats. Individual colours represent distinct biological repeats. *** *p <* 0.001, * *p <* 0.05. One-way ANOVA with Dunnett’s *post hoc* test. Scale bar, 100 μm.


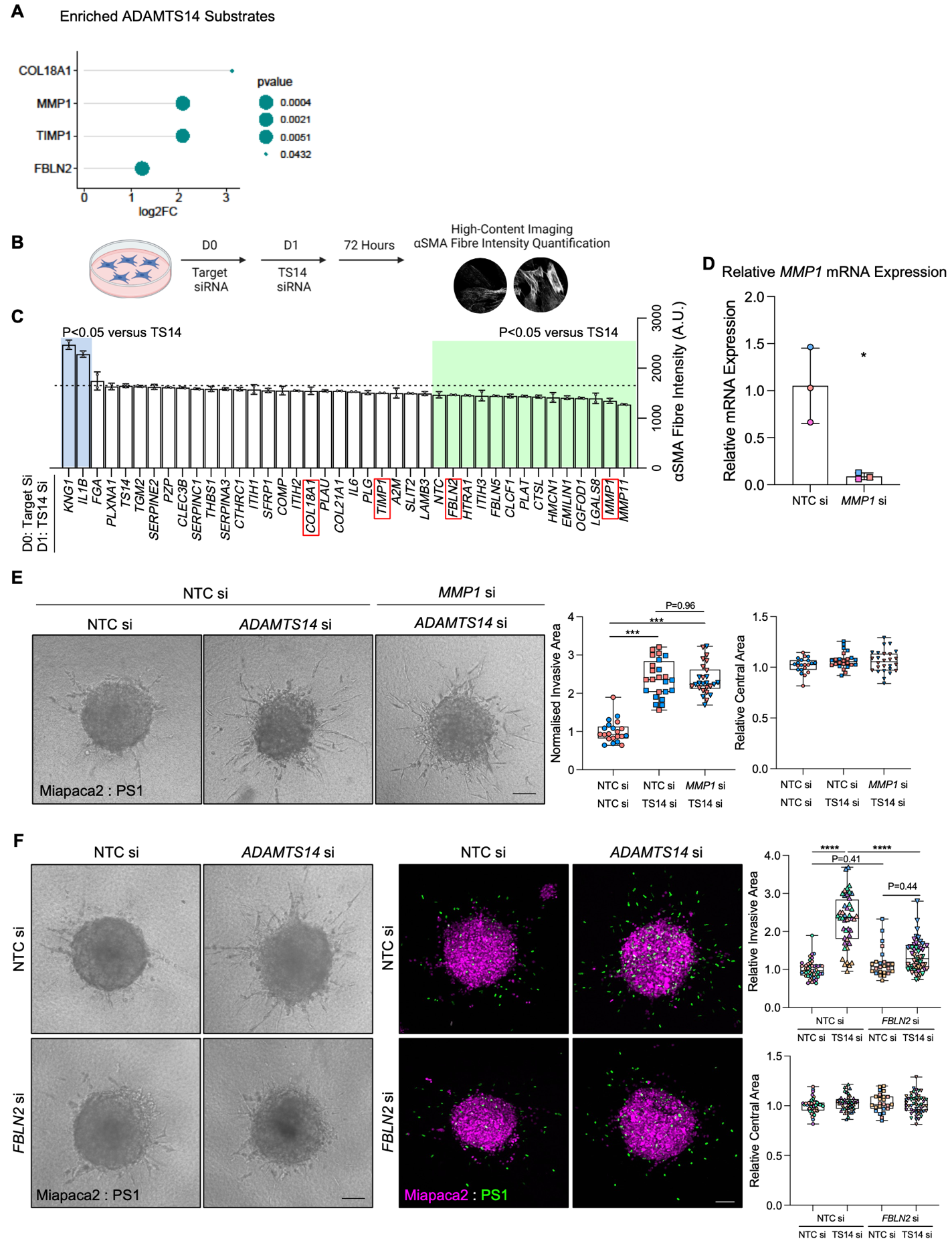


**Figure S5.** **siRNA screen reveals fibulin 2 as a mediator of ADAMTS14 function.** (A) Lollipop plot of enriched ADAMTS14 substrates from matrisome data. (B) Schematic of high-content siRNA screen. (C) αSMA expression in stellate cells with knockdown of *ADAMTS14* and co-knockdown of the indicated gene product. Expression of siRNAs that cause an αSMA intensity significantly different from *ADAMTS14* knockdown alone are highlighted in blue and green. Known ADAMTS14 substrates are highlighted in red. Numbers representative of two biological replicates each performed in duplicate wells. (D) RT-qPCR for *MMP1* expression in human PS1 stellate cells following siRNA knockdown of *MMP1*. (E) Brightfield images and quantification of invasion and central area from Miapaca2 cancer cells: human PS1 stellate cell spheroids with siRNA knockdown of *ADAMTS14* (TS14) with and without co-knockdown of *MMP1*. (F) Brightfield and confocal images and quantification of invasion and central area from miapaca2 (H2B-RFP, purple): human PS1 stellate cell (H2B-GFP, green) spheroids with siRNA knockdown of *ADAMTS14* with and without co-knockdown of *FBLN2*. Scale bar, 100 μm. Images representative of at least two biological repeats. Individual colours represent distinct biological repeats. **** *p <* 0.0001, *** *p <* 0.001. One-way ANOVA with Dunnett’s *post hoc* test.

**Supplementary Tables S1,S2 are provided as separate Excel files**

**Table S3.** Primer sequences used.

| Target Gene | Forward Primer | Reverse Primer |
| --- | --- | --- |
| Human *ADAMTS2* | GTCCCCGAAACATCTCAGATC | GGACAAGACTTCCATCCTACAG |
| Human *MMP1* | ACAGCCCAGTACTTATTCCCTTTG | GGGCTTGAAGCTGCTTACGA |
| Human *ACTB* | AGAGCTACGAGCTGCCTGAC | AGCACTGTGTTGGCGTACAG |
| Mouse *Serpine2* | AGAATGTGAACTTCCAGGACC | ACACTGCATTAACGAGGACC |
| Mouse *Adamts2* | GATAATTTTGGTGTCTGCCGG | TCTGGATAGGTGAGTCAGGATC |
| Mouse *Timp1* | CTCAAAGACCTATAGTGCTGGC | CAAAGTGACGGCTCTGGTAG |
| Mouse *Actb* | ACCTTCTACAATGAGCTGCG | CTGGATGGCTACGTACATGG |
